# The Reconstructed Individual Patient Data from Kaplan–Meier (IPDfromKM) Method for Non-Inferiority Analyses: A New Potential Application

**DOI:** 10.3390/mps8010013

**Published:** 2025-02-02

**Authors:** Eugenia Piragine, Sabrina Trippoli, Sara Veneziano, Andrea Messori, Vincenzo Calderone

**Affiliations:** 1 Department of Pharmacy, University of Pisa, Via Bonanno 6, 56126 Pisa, Italy; sara.veneziano@phd.unipi.it (S.V.); vincenzo.calderone@unipi.it (V.C.); 2 School of Specialization in Hospital Pharmacy, University of Pisa, Via Bonanno 6, 56126 Pisa, Italy; 3 HTA Unit, Centro Operativo, Regione Toscana, Via Alderotti 26/N, 50141 Firenze, Italy; sabrina.trippoli@estar.toscana.it (S.T.); andrea.messori@regione.toscana.it (A.M.); 4 Comitato Scientifico SIFaCT, Società Italiana di Farmacia Clinica e Terapia, Via Guarini 4, 10123 Torino, Italy

**Keywords:** IPDfromKM method, Shiny method, non-inferiority analysis, atrial appendage occlusion devices, anticoagulants, atrial fibrillation

## Abstract

The IPDfromKM method, or Shiny method, is an artificial intelligence tool that enables indirect comparisons between studies by reconstructing individual patient data (IPD) from Kaplan–Meier (KM) curves. The IPDfromKM method is generally used for superiority analyses, but a further application could be represented by non-inferiority analyses. However, there are no studies supporting this methodological hypothesis. The aim of this work was to validate the IPDfromKM method for non-inferiority analyses by “exploiting” the well-described non-inferiority of implantable devices occluding the left atrial appendage compared to oral anticoagulants in patients with atrial fibrillation. We performed a systematic review searching for randomized controlled trials (RCTs) in the PubMed database and found five studies. The R software (version 4.3.3) was used to perform a standard survival analysis comparing Watchman and Amlet devices with warfarin. The hazard ratio (HR), with 95% confidence interval (CI), was the main parameter of our analysis. The results confirmed the non-inferiority of Amlet and Watchman compared to warfarin (HR of Watchman vs. warfarin: 1.23, 95% CI 0.80 to 1.9; HR of Amlet vs. warfarin: 1.05, 95% CI 0.61 to 1.80). Therefore, we proposed a new application of the IPDfromKM method that could be potentially relevant in decision-making for the management of this common cardiac arrhythmia and a wide range of other pathological conditions.

## 1. Introduction

**Scheme 1:**
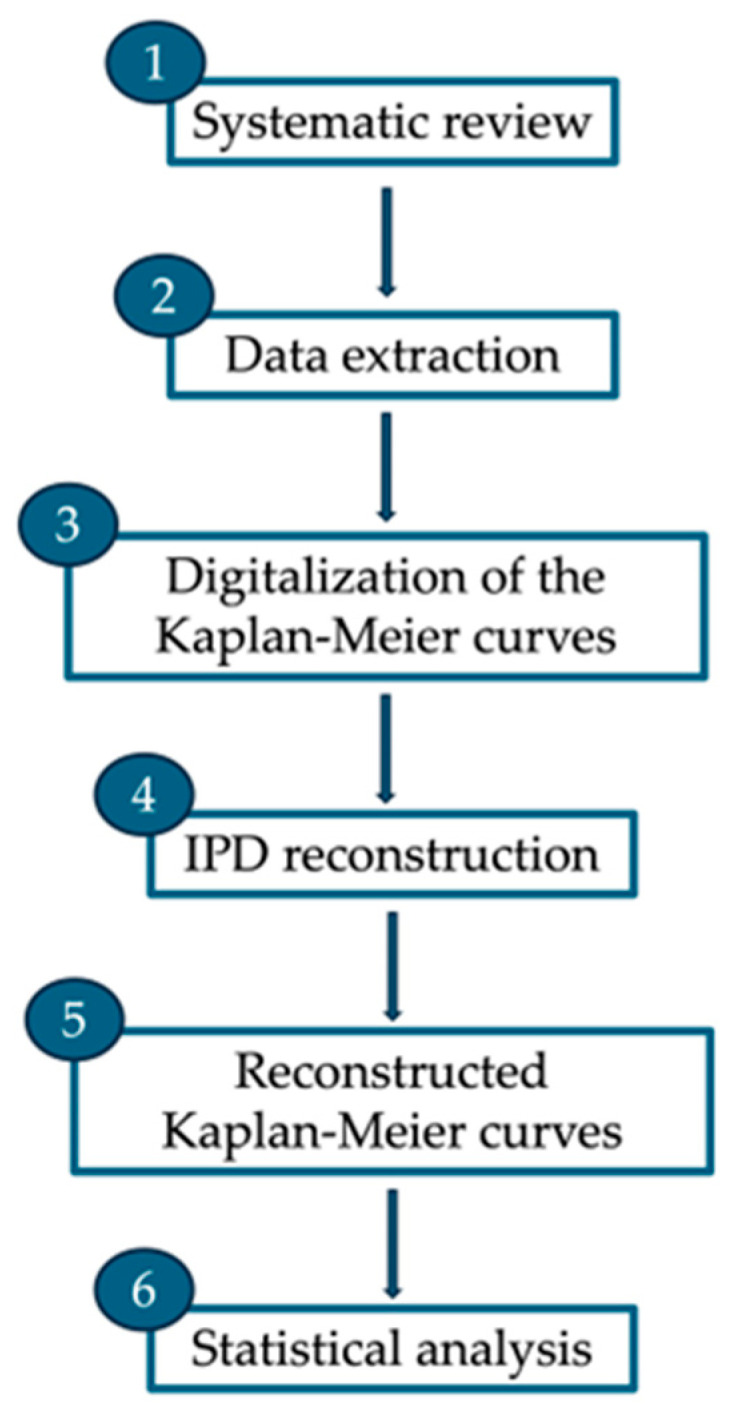
Main steps of the IPDfromKM method.

The IPDfromKM method, or Shiny method, is an innovative tool that uses artificial intelligence (AI) to reconstruct individual patient data (IPD) from Kaplan–Meier (KM) curves [1,2,3,4,5,6]. This method was first proposed in 2012 [5] and validated in 2022 by Rogula and colleagues, who demonstrated a perfect overlap between the original and digitized/reconstructed KM curves [7]. This method provides access to IPD that can be useful in clinical practice and allows for indirect comparisons between studies based on reconstructed patients to generate original clinical evidence. The IPDfromKM method ensures a more appropriate analysis of time-to-event endpoints, especially those with a long follow-up, than a standard binary meta-analysis because it considers the time at which events occur and adjusts the analysis based on the presence of censored patients. In addition, the results are presented in a very intuitive way by generating a single survival graph based on the reconstructed patients, in which all patients who received the same treatment are combined into a single KM curve. Therefore, the KM graph contains as many KM curves as there are treatments to be compared. Standard statistical methods (e.g., hazard ratio [HR], log-rank test, etc.) can be used to analyze the reconstructed patient curves considering the time-course of the entire follow-up [1,2,3,4,5,6,7]. For these reasons, the IPDfromKM method can overcome some limitations of the standard binary meta-analysis in performing indirect comparisons of time-to-event endpoints. Scheme 1 provides a schematic representation summarizing the main steps of the IPDfromKM method.

In recent years, the IPDfromKM method has been used by an increasing number of studies to perform superiority analyses, confirming the interest from the scientific community in this tool [3,8,9,10,11,12,13]. However, there are no current studies that support the potential application of the IPDfromKM method in non-inferiority analyses as well. Therefore, this article focused on a well-known non-inferiority comparison between medical devices and drug therapy in patients with atrial fibrillation [14] to validate the IPDfromKM method for non-inferiority studies, thus strengthening the methodological value of this statistical technique and extending its scope of application.

Atrial fibrillation is a common cardiac arrhythmia and a major cause of stroke and heart failure worldwide [15,16,17]. Chronic warfarin therapy has long been the first-line treatment for patients with atrial fibrillation, but the need for regular monitoring of prothrombin time and international normalized ratio (INR), which negatively impacts patient compliance, has led to the development of novel oral anticoagulants (NOACs) [18,19]. These drugs have numerous advantages over warfarin, such as reduced drug interactions and stable dosing regimen [20,21]. However, chronic use of warfarin and NOACs is often associated with increased bleeding risk and suboptimal patient adherence [18,22]. Therefore, the identification of effective alternatives to chronic anticoagulant therapy is essential to ensure better clinical management of atrial fibrillation in non-adherent or non-responsive patients. Several reports and numerous registry-based observational studies have been published on the efficacy and safety of implantable devices that occlude the left atrial appendage. Proprietary names include Amlet (also called ACP) and Watchman (available in three versions called Watchman, Watchman 2.5, and Watchman FLX) [23,24], whose non-inferiority to oral anticoagulant therapy in patients with atrial fibrillation was recently confirmed in a systematic review combined with a standard network meta-analysis of seven randomized controlled trials (RCTs) [14]. In this work, Oliva and colleagues demonstrated that there was no significant difference in the risk of stroke or systemic embolism between implantable devices occluding the left atrial appendage and vitamin K antagonists or NOACs; however, non-inferiority statistics were not performed.

The aim of our study was to validate the IPDfromKM method for non-inferiority analyses using this well-known comparison (i.e., implantable devices occluding the left atrial appendage vs. traditional drugs) as a reference. This could allow us to present a new application of the IPDfromKM technique and provide researchers and clinicians with an optimized tool for secondary analyses of survival data based on a non-inferiority design.

## 2. Materials and Methods

### 2.1. Study Selection

This systematic review was conducted by searching the Medline database (via PubMed) to identify studies evaluating the efficacy of atrial appendage occlusion devices and oral anticoagulant drugs in patients with atrial fibrillation (last searched on 5 April 2024). The following search terms were used: “atrial appendage occlusion” AND “(Watchman OR Amlet)”. The study selection process was conducted by two authors (S.T. and A.M.).

### 2.2. Inclusion Criteria

Inclusion criteria were (i) patients at risk of death, stroke or systemic embolism of cardiovascular origin; (ii) use of anticoagulants or implantation of appendage occlusion devices (Amlet or Watchman); (iii) endpoint defined as a composite of cardiovascular death, stroke, or systemic embolism; (iv) the publication of results as a KM curve of event-free survival; and (v) RCTs, searched by applying the specific filter option to directly compare our results with those of previous “traditional” meta-analyses on the same topic, in which only RCTs were selected [14]. For each study, reasons for inclusion and exclusion were recorded. When two or more publications focused on the same trial, only the most recent was included.

### 2.3. Exclusion Criteria

Non-randomized studies were excluded because they are subject to important biases such as the different inclusion criteria, ranging from enrolling consecutive patients to selecting subgroups of patients with specific characteristics.

### 2.4. Data Extraction

For each study, the number of enrolled patients, patient characteristics and the number of events based on the composite endpoint of cardiovascular death, stroke, or systemic embolism were extracted by two authors (E.P. and S.V.) in a Microsoft Excel spreadsheet (version 2022). The disputes were settled with a third author (A.M.).

### 2.5. Reconstruction of Individual Patient Data (IPD)

The IPDfromKM method was used to reconstruct IPD from survival curves [5,7]. The curves were digitized with Webplotdigitizer (version 4.5; accessed on 10 January 2024) and then inserted into the IPD reconstruction tool of the IPDfromKM software (version 1.2.3.0; subprogram “Reconstruct Individual Patient Data”). The reconstructed IPD included the time of observation (i.e., difference between enrollment and last follow-up) and the patient’s outcome at the last follow-up (alive, dead, or censored).

### 2.6. Statistical Analysis

Restricted mean survival time (RMST) [25] and event-free survival were estimated using Cox statistics for time-to-event endpoints (i.e., composite of cardiovascular death, stroke, or systemic embolism); medians with 95% CI and HRs with 95% confidence interval (CI) were calculated. The heterogeneity between studies was assessed by likelihood ratio test while indirect head-to-head comparisons between treatments were performed using four packages of the R-platform (version 4.2.1), according to the Cox model: ‘survival’, ‘survRM2’, ‘survminer’, and ‘ggsurvplot’ (2020; https://www.R-project.org/, accessed on 18 December 2023).

### 2.7. Non-Inferiority Analysis

First, we identified the non-inferiority margin applied to the composite endpoint and expressed according to the RMST. This non-inferiority margin, based on the primary endpoint of our analysis, was set at a loss not exceeding 2 months without events over a 30-month follow-up. Then, we evaluated whether the non-inferiority of the two devices vs. anticoagulants was achieved by a Forest plot that presents both the margin and incremental benefit (or loss of time-to-event survival) with 90% CI for the two devices. The use of the 90% CI, instead of 95% CI, in non-inferiority studies is explained in the article by Walker and Novacki [26].

## 3. Results

### 3.1. Systematic Review

**Figure 1:**
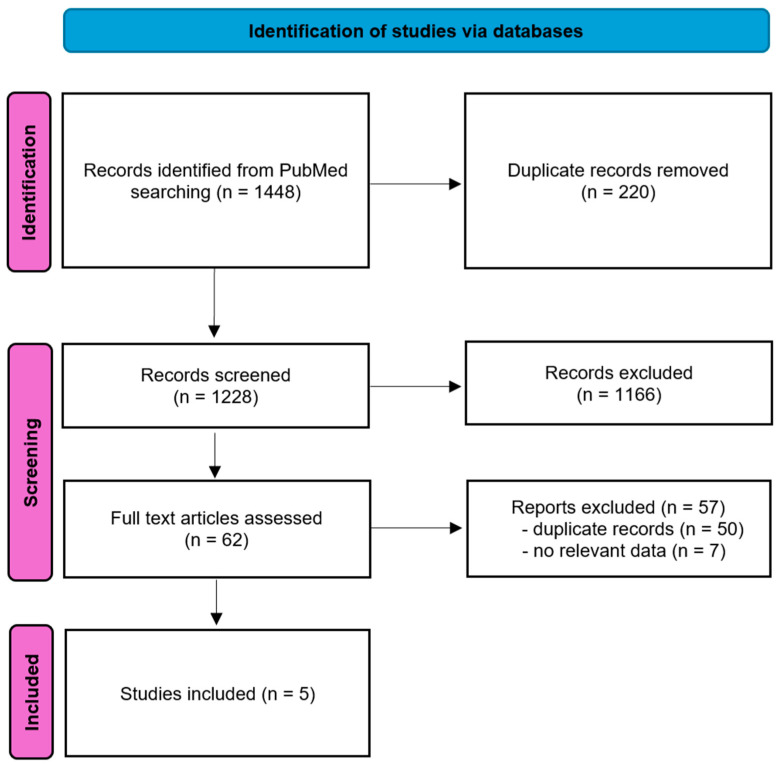
PRISMA flow diagram.

The study selection process is summarized in Figure 1. A total of 1448 records were identified via Medline. After the removal of duplicates, 1228 records were examined for eligibility. Then, 1166 were excluded and 62 full texts were recovered. Fifty studies were excluded as they were duplicated and seven because they did not present data relevant to our analysis. Therefore, 5 RCTs [27,28,29,30,31] were included in the systematic review.

The main characteristics of the 5 studies are shown in Table 1. There were 1783 patients who received the Watchman implant, 1054 who received the Amlet implant, and 382 who received drug therapy (warfarin). All patients had a similar disease severity, age and gender except in the study by Lakireddy and colleagues [29], where the proportion of men was slightly lower. The total number of events was 221 (Table 2).

### 3.2. Reconstruction of IPD

**Figure 2:**
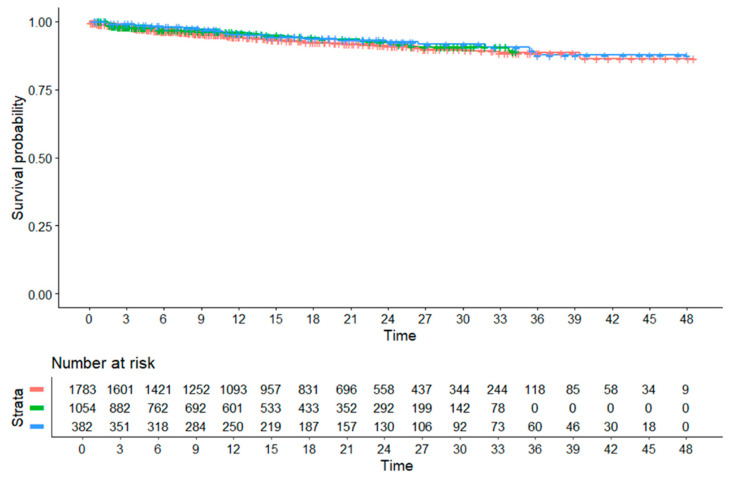
The reconstruction of individual patient data (IPD) from KM curves using the IPDfromKM method. Colour lines: red, Watchman; green, Amlet; blue, warfarin. Time in months.

The reconstructed KM curves are shown in Figure 2. A comparison between Watchman vs. warfarin gave an HR of 1.2324 (95% CI: 0.796 to 1.907), between Amlet vs. warfarin gave an HR of 1.05 (95% CI: 0.614 to 1.796) and between Amlet vs. Watchman gave an HR of 0.8525 (95% CI: 0.6244 to 1.164). In the overall analysis, the heterogeneity level between studies was not statistically significant (likelihood ratio test = 1.58 on 2 df, *p* = 0.50 and Wald’s test = 1.55 on 2 df, *p* = 0.50).

### 3.3. Non-Inferiority Analysis

The RMST values based on a 30-month truncation time and estimated for the pooled patient groups of the three treatments are shown in Table 3. These values were very similar to each other and, therefore, all incremental benefits (or survival gains) expressed as a gain in event-free months were particularly small (and vice versa, all losses in the months without events). The incremental benefit of warfarin vs. Amlet was 0.136 event-free months (95% CI: −0.579 to 0.85; 90% CI: −0.464 to 0.735), while for warfarin vs. Watchman, this was 0.162 (95% CI: −0.714 to 1.038; 90% CI: −0.573 to 0.897). The loss in RMST for both devices compared to warfarin can be easily calculated by inverting the algebraic sign of the above benefits and converting 95% CI to 90% CI.

**Figure 3:**
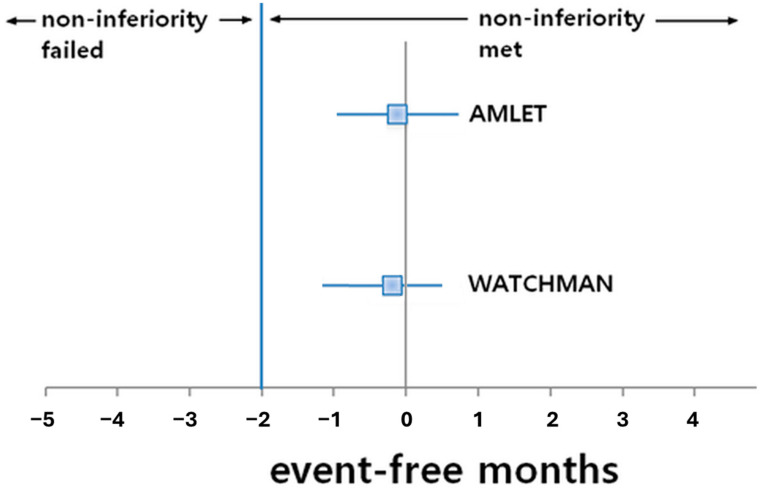
Non-inferiority assessment vs. warfarin.

The results of the non-inferiority analysis are summarized in Figure 3. The Forest plot shows that CIs have remained far from the non-inferiority margin (set at a 2-month loss in event-free survival, as indicated above). Hence, non-inferiority has been clearly demonstrated for both devices (p_non-inferiority_ < 0.01 for Amlet and <0.05 for Watchman).

## 4. Discussion

In this study, the non-inferiority of Amlet and Watchman to warfarin was confirmed using the innovative IPDfromKM method [1]. No significant difference in risk was found for the composite endpoint (cardiovascular death, stroke or systemic embolism) in patients with atrial fibrillation who have an atrial occlusion device implanted compared to those using warfarin. In addition, the survival model of reconstructed patients showed that survival gains, expressed as event-free months, were similar for all treatments, confirming the non-inferiority of both medical devices to drug therapy. Finally, in our non-inferiority analysis, the statistical approach (including RMST and use of the 90% CI) was consistent with current standards for this type of statistic. The non-inferiority margin (set at a 2-month loss over 30 months) reflected a reasonable clinical decision and, as always, was based purely on expert opinion for the specific problem studied. However, the statistical validity of this non-inferiority test was confirmed by the significant values of p_non-inferiority_.

This is the first study in which IPDfromKM has been applied to perform a non-inferiority analysis, leading to conclusions similar to those previously obtained with “classical” methodologies (e.g., meta-analysis) [14]. From a methodological point of view, this study validated the IPDfromKM method as a reliable and valid method not only for superiority analyses, but also for non-inferiority analyses. Clinically, our results have reinforced the important role of atrial appendage closure in patients with atrial fibrillation and have allowed us to conclude that Amlet or Watchman are non-inferior to warfarin, a result that provides better information than a non-significant difference.

The main strength of this study is the originality of the method applied, which combines IPDfromKM with the complex principles of a non-inferiority analysis. Our research group is currently conducting an analysis using similar methods to assess the non-inferiority of transcatheter valve replacement and surgery [32].

An important limitation of this study is the selection of a single non-inferiority example for method validation. We are aware that further non-inferiority examples are needed to confirm the validity of the method and improve the generalizability of the IPDfromKM technique. However, presenting more than one systematic review here could be counterproductive, as it could lead to misleading or confusing results. Therefore, the application of the IPDfromKM method for further non-inferiority analyses could be a future direction of this work.

Another aspect to consider is the potential loss of some efficacy data due to the decision to include only studies with KM curves. Furthermore, although inclusion criteria based solely on studies with randomized design gave homogeneity to the five RCTs included in the analysis, five studies are objectively few. This is a common limitation of studies examining the efficacy of left atrial appendage closure devices in patients with atrial fibrillation (see, for example, the meta-analysis of seven studies by Oliva and colleagues [14]). To increase the number of studies, we could have selected other study designs, such as non-randomized controlled trials, but we preferred not to include this potential source of bias in our analysis. In fact, the purpose of our study was not to provide new evidence on the non-inferiority of Watchman or Amlet compared to oral anticoagulants, but to validate the IPDfromKM for non-inferiority analyses. Therefore, we decided not to include study designs other than RCTs. On the other hand, it is worth mentioning that some important non-randomized studies were published on the Watchman and Amlet devices but not included because a randomized design was missing. Two of these are mainly relevant because they included many single-arm studies or registries [33,34], while three of these studies are ongoing [35,36,37] and have not been included because their results are not yet available. At the same time, we are aware that the exclusion of high-quality observational studies may have led to the loss of potentially useful data. Indeed, while they are associated with lower methodological quality and hence internal validity compared to RCTs, these studies may be more informative than RCTs due to the greater generalizability of results, the longer follow-up, and the possibility of identifying rare adverse events.

## 5. Conclusions

In this study, we proposed the use of the IPDfromKM method for non-inferiority analyses and suggested a further application of this statistical technique for indirect comparisons of time-to-event endpoint. Our results support the clinical use of atrial appendage occlusion devices in patients with atrial fibrillation as an effective and non-inferior alternative to chronic warfarin therapy. In addition, they confirm the potential role of the IPDfromKM technique in decision-making for the management of various pathological conditions, including atrial fibrillation. Based on these findings, cardiologists may consider replacing anticoagulant therapy with atrial appendage occlusion devices in “selected” patients. However, future RCTs and head-to-head comparisons are needed to confirm these preliminary findings and the validity/reproducibility of the method. Also, since all the included studies have focused on warfarin, the investigation of the non-inferiority of atrial appendage occlusion devices compared with NOACs could be another future direction of this work. In fact, the only randomized clinical trial (PRAGUE-17) designed to compare the efficacy of atrial appendage occlusion devices with NOACs in preventing stroke, systemic embolism, and cardiovascular death in patients with atrial fibrillation did not report data on Watchman and Amlet separately [38] and, therefore, could not be included in our analysis. It is important to note that the results of the PRAGUE-17 trial confirmed those of our study, as the appendage occlusion devices were not inferior to NOACs in preventing the composite endpoint.

## Figures and Tables

**Table 1:** The main characteristics of the included RCTs. Abbreviations: AF, atrial failure; NVAF, nonvalvular atrial failure; SD: standard deviation.

First Author, Reference	Patient Characteristics	Comparators	No. of Patients (A)	No. of Patients (B)	Age, Years ± SD (A)	Age, Years ± SD (B)	Men, % (A)	Men, % (B)
Galea [27]	High-risk AF patients with clinical indication to atrial appendage occlusion devices	Amlet (A) vs. Watchman (B)	111	110	76.5 ± 7.1	77.3 ± 8.4	71.2	70.0
Lakkireddy [29]	Adult patients with paroxysmal, persistent, or permanent NVAF and at high risk of stroke or systemic embolism (CHADS_2_ score ≥ 2 or CHA_2_DS_2_-VASc score ≥ 3)	Amlet (A) vs. Watchman (B)	917	916	75.0 ± 7.6	75.2 ± 7.6	58.6	61.4
Mansour [30]	Patients with a mean CHA_2_DS_2_-VASc score 3.94 ± 1.2 underwent left atrial appendage occlusion	Amlet (A) vs. Watchman (B)	26	25	75 ± 7.4	76 ± 6.9	76.0	76.0
Holmes [28]	Patients with NVAF (paroxysmal, persistent, or permanent) with CHADS_2_ score ≥ 2 or 1 and another risk factor	Watchman (A) vs. warfarin (B)	269	138	74.0 ± 7.4	74.9 ± 7.2	74.6	67.7
Reddy [31]	Adult patients with a history of paroxysmal, persistent, or permanent NVAF plus at least 1 additional stroke risk factor, eligible for warfarin therapy	Watchman (A) vs. warfarin (B)	463	244	-	-	-	-

**Table 2:** The main results of the RCTs included. Abbreviations: CI, confidence interval; HR, hazard ratio.

First Author, Reference	Comparators	No. of Events/Total No. of Patients			Published HR (95% CI)
		Amlet	Watchman	Warfarin	
Galea [27]	Amlet vs. Watchman	10/111	11/110	-	0.91 (0.39 to 2.14)
Lakkireddy [29]	Amlet vs. Watchman	95/917	105/916	-	0.87 (0.66 to 1.14)
Mansour [30]	Amlet vs. Watchman	0/26	6/25	-	Not reported; *p* = 0.008
Holmes [28]	Watchman vs. warfarin	-	14/269	4/138	1.07 (0.57 to 1.89)
Reddy [31]	Watchman vs. warfarin	-	31/463	24/244	0.71 (0.44 to 1.30)

**Table 3:** Restricted mean survival time (RMST) of the pooled patient groups.

Intervention	RMST (Months)	95% CI
Watchman	28.146	27.826 to 28.466
Amlet	28.443	28.049 to 28.838
Warfarin	28.579	27.983 to 29.175

## Data Availability

Data are available on request from the corresponding author.

## References

[1] Liu N., Zhou Y., Lee J.J. (2021). IPDfromKM: Reconstruct individual patient data from published Kaplan-Meier survival curves. BMC Med. Res. Methodol..

[2] Messori A. (2024). Reconstruction of individual-patient data from the analysis of Kaplan-Meier curves: This method is widely used in oncology and cardiology—List of 53 studies in oncology and 61 in cardiology (preprint). Open Sci. Framew..

[3] Zhang S., Li S., Cheng Y. (2024). The efficacy and safety of immunotherapy as first-line treatment for extensive-stage small cell lung cancer: Evaluating based on reconstructed individual patient data. Front. Oncol..

[4] Wang B.C., Fu C., Lin G.H. (2023). The efficacy of adebrelimab compared with durvalumab and atezolizumab in untreated extensive-stage small-cell lung cancer: A survival analysis of reconstructed patient-level data. Front. Immunol..

[5] Guyot P., Ades A.E., Ouwens M.J., Welton N.J. (2012). Enhanced secondary analysis of survival data: Reconstructing the data from published Kaplan-Meier survival curves. BMC Med. Res. Methodol..

[6] Wei Y., Royston P. (2017). Reconstructing time-to-event data from published Kaplan-Meier curves. Stata J..

[7] Rogula B., Lozano-Ortega G., Johnston K.M. (2022). A Method for Reconstructing Individual Patient Data From Kaplan-Meier Survival Curves That Incorporate Marked Censoring Times. MDM Policy Pract..

[8] Piragine E., Veneziano S., Trippoli S., Messori A., Calderone V. (2024). Efficacy and Safety of Cardioband in Patients with Tricuspid Regurgitation: Systematic Review and Meta-Analysis of Single-Arm Trials and Observational Studies. J. Clin. Med..

[9] Ossato A., Gasperoni L., Del Bono L., Messori A., Damuzzo V. (2024). Efficacy of Immune Checkpoint Inhibitors vs. Tyrosine Kinase Inhibitors/Everolimus in Adjuvant Renal Cell Carcinoma: Indirect Comparison of Disease-Free Survival. Cancers.

[10] Peng T.R., Wu T.W., Yi T.Y., Wu A.J. (2024). Comparative Efficacy of Adagrasib and Sotorasib in KRAS G12C-Mutant NSCLC: Insights from Pivotal Trials. Cancers.

[11] Sá M.P., Van den Eynde J., Jacquemyn X., Tasoudis P., Erten O., Dokollari A., Torregrossa G., Sicouri S., Ramlawi B. (2023). Late outcomes of transcatheter aortic valve implantation in bicuspid versus tricuspid valves: Meta-analysis of reconstructed time-to-event data. Trends Cardiovasc. Med..

[12] Wach J., Vychopen M., Güresir A., Guranda A., Nestler U., Güresir E. (2024). A Long-Term Comparative Analysis of Endovascular Coiling and Clipping for Ruptured Cerebral Aneurysms: An Individual Patient-Level Meta-Analysis Assessing Rerupture Rates. J. Clin. Med..

[13] Cashman K.D., Ritz C. (2019). Individual participant data (IPD)-level meta-analysis of randomised controlled trials among dark-skinned populations to estimate the dietary requirement for vitamin D. Syst. Rev..

[14] Oliva A., Ioppolo A.M., Chiarito M., Cremonesi A., Azzano A., Micciche E., Mangiameli A., Ariano F., Ferrante G., Reimers B. (2024). Left Atrial Appendage Closure Compared with Oral Anticoagulants for Patients with Atrial Fibrillation: A Systematic Review and Network Meta-Analysis. J. Am. Heart Assoc..

[15] Saleh A., Haldar S. (2023). Atrial fibrillation: A contemporary update. Clin. Med..

[16] Elsheikh S., Hill A., Irving G., Lip G.Y.H., Abdul-Rahim A.H. (2024). Atrial fibrillation and stroke: State-of-the-art and future directions. Curr. Probl. Cardiol..

[17] Essa H., Hill A.M., Lip G.Y.H. (2021). Atrial Fibrillation and Stroke. Card. Electrophysiol. Clin..

[18] Salmasi S., Loewen P.S., Tandun R., Andrade J.G., De Vera M.A. (2020). Adherence to oral anticoagulants among patients with atrial fibrillation: A systematic review and meta-analysis of observational studies. BMJ Open.

[19] Spruit J.R., de Vries T.A.C., Hemels M.E.W., Pisters R., de Groot J.R., Jansen R.W.M.M. (2024). Direct Oral Anticoagulants in Older and Frail Patients with Atrial Fibrillation: A Decade of Experience. Drugs Aging.

[20] Xu K., Chan N.C., Eikelboom J.W. (2021). Strategies for the prevention and treatment of bleeding in patients treated with dabigatran: An update. Expert Opin. Drug Metab. Toxicol..

[21] Silverio A., Di Maio M., Prota C., De Angelis E., Radano I., Citro R., Carrizzo A., Ciccarelli M., Vecchione C., Capodanno D. (2021). Safety and efficacy of non-vitamin K antagonist oral anticoagulants in elderly patients with atrial fibrillation: Systematic review and meta-analysis of 22 studies and 440 281 patients. Eur. Heart J. Cardiovasc. Pharmacother..

[22] Ballestri S., Romagnoli E., Arioli D., Coluccio V., Marrazzo A., Athanasiou A., Di Girolamo M., Cappi C., Marietta M., Capitelli M. (2023). Risk and Management of Bleeding Complications with Direct Oral Anticoagulants in Patients with Atrial Fibrillation and Venous Thromboembolism: A Narrative Review. Adv. Ther..

[23] Bing S., Chen R.R. (2023). Clinical efficacy and safety comparison of Watchman device versus ACP/Amulet device for percutaneous left atrial appendage closure in patients with nonvalvular atrial fibrillation: A study-level meta-analysis of clinical trials. Clin. Cardiol..

[24] Ueno H., Imamura T., Tanaka S., Fukuda N., Kinugawa K. (2023). Left atrial appendage closure for stroke prevention in nonvalvular atrial fibrillation: A current overview. J. Cardiol..

[25] Damuzzo V., Agnoletto L., Leonardi L., Chiumente M., Mengato D., Messori A. (2019). Analysis of Survival Curves: Statistical Methods Accounting for the Presence of Long-Term Survivors. Front. Oncol..

[26] Walker E., Nowacki A.S. (2011). Understanding equivalence and noninferiority testing. J. Gen. Intern. Med..

[27] Galea R., Meneveau N., De Marco F., Aminian A., Heg D., Chalkou K., Grani C., Anselme F., Franzone A., Vranckx P. (2024). One-Year Outcomes After Amulet or Watchman Device for Percutaneous Left Atrial Appendage Closure: A Prespecified Analysis of the SWISS-APERO Randomized Clinical Trial. Circulation.

[28] Holmes D.R., Kar S., Price M.J., Whisenant B., Sievert H., Doshi S.K., Huber K., Reddy V.Y. (2014). Prospective randomized evaluation of the Watchman Left Atrial Appendage Closure device in patients with atrial fibrillation versus long-term warfarin therapy: The PREVAIL trial. J. Am. Coll. Cardiol..

[29] Lakkireddy D., Thaler D., Ellis C.R., Swarup V., Gambhir A., Hermiller J., Nielsen-Kudsk J.E., Worthley S., Nair D., Schmidt B. (2023). 3-Year Outcomes From the Amplatzer Amulet Left Atrial Appendage Occluder Randomized Controlled Trial (Amulet IDE). JACC Cardiovasc. Interv..

[30] Mansour M.J., Harnay E., Al Ayouby A., Mansourati V., Jobic Y., Gilard M., Le Ven F., Mansourati J. (2022). One year outcome and analysis of peri-device leak of left atrial appendage occlusion devices. J. Interv. Card. Electrophysiol..

[31] Reddy V.Y., Doshi S.K., Sievert H., Buchbinder M., Neuzil P., Huber K., Halperin J.L., Holmes D., Investigators P.A. (2013). Percutaneous left atrial appendage closure for stroke prophylaxis in patients with atrial fibrillation: 2.3-Year Follow-up of the PROTECT AF (Watchman Left Atrial Appendage System for Embolic Protection in Patients with Atrial Fibrillation) Trial. Circulation.

[32] Messori A., Trippoli S., Fadda V. (2024). Transcatheter Versus Surgical Aortic Valve Replacement in Patients at Low Risk: Long-Term Outcomes From the Three Pivotal Randomised Trials Determined by Reconstruction of Individual Patient Data From Kaplan-Meier Curves. Heart Lung Circ..

[33] Panikker S., Lord J., Jarman J.W., Armstrong S., Jones D.G., Haldar S., Butcher C., Khan H., Mantziari L., Nicol E. (2016). Outcomes and costs of left atrial appendage closure from randomized controlled trial and real-world experience relative to oral anticoagulation. Eur. Heart J..

[34] Price M.J., Reddy V.Y., Valderrabano M., Halperin J.L., Gibson D.N., Gordon N., Huber K.C., Holmes D.R. (2015). Bleeding Outcomes After Left Atrial Appendage Closure Compared with Long-Term Warfarin: A Pooled, Patient-Level Analysis of the WATCHMAN Randomized Trial Experience. JACC Cardiovasc. Interv..

[35] Korsholm K., Damgaard D., Valentin J.B., Packer E.J.S., Odenstedt J., Sinisalo J., Putaala J., Naess H., Al-Jazi M.A., Karlsson J.E. (2022). Left atrial appendage occlusion vs novel oral anticoagulation for stroke prevention in atrial fibrillation: Rationale and design of the multicenter randomized occlusion-AF trial. Am. Heart J..

[36] Osmancik P., Herman D., Neuzil P., Hala P., Taborsky M., Kala P., Poloczek M., Stasek J., Haman L., Branny M. (2022). 4-Year Outcomes After Left Atrial Appendage Closure Versus Nonwarfarin Oral Anticoagulation for Atrial Fibrillation. J. Am. Coll. Cardiol..

[37] Wazni O.M., Boersma L., Healey J.S., Mansour M., Tondo C., Phillips K., Doshi R., Jaber W., Hynes E., Allocco D.J. (2022). Comparison of anticoagulation with left atrial appendage closure after atrial fibrillation ablation: Rationale and design of the OPTION randomized trial. Am. Heart J..

[38] Osmancik P., Herman D., Neuzil P., Hala P., Taborsky M., Kala P., Poloczek M., Stasek J., Haman L., Branny M. (2020). Left Atrial Appendage Closure Versus Direct Oral Anticoagulants in High-Risk Patients with Atrial Fibrillation. J. Am. Coll. Cardiol..

